# Clinical Utility of Plasma Microbial Cell-Free DNA Surveillance in Neutropenic Patients with Acute Myeloid Leukemia Undergoing Outpatient Chemotherapy: A Case Series

**DOI:** 10.3390/diagnostics15131715

**Published:** 2025-07-05

**Authors:** Maria Lampou, Elizabeth C. Trull, Hailey M. Warren, Musie S. Ghebremichael, Raja Nakka, Daniel J. Floyd, Amir T. Fathi, Andrew M. Brunner, Michael K. Mansour

**Affiliations:** 1 Division of Infectious Diseases, Massachusetts General Hospital, Boston, MA 02114, USA; mlampou@mgh.harvard.edu (M.L.); etrull@mgh.harvard.edu (E.C.T.); hmwarren@mgb.org (H.M.W.); djf58@duke.edu (D.J.F.); 2 Department of Medicine, Harvard Medical School, Boston, MA 02115, USA; 3 Ragon Institute of Mass General Brigham, Massachusetts Institute of Technology and Harvard, Cambridge, MA 02139, USA; rnakka@mgh.harvard.edu; 4 Massachusetts General Hospital Biostatistics, Boston, MA 02114, USA; 5 Department of Hematology/Oncology, Massachusetts General Hospital, Boston, MA 02114, USA

**Keywords:** microbial cell-free DNA, infections, hematologic malignancies, acute myeloid leukemia, neutropenia, immunocompromised, outpatient, chemotherapy

## Abstract

**Background/Objectives**: The main objective of the study is to assess the clinical utility of microbial cell-free DNA (mcfDNA) in neutropenic patients diagnosed with acute myeloid leukemia (AML) undergoing chemotherapy in the outpatient setting. Neutropenia is a common complication in this patient cohort and enhances the risk of fatal opportunistic bacterial and fungal infections. Accurate and timely diagnosis of these infections in outpatient asymptomatic individuals is critical. **Methods**: Fourteen patients were studied in this prospective observational case series. Traditional blood cultures (BCs) were obtained when clinically indicated and blood samples were collected for plasma mcfDNA metagenomic sequencing up to two times a week at outpatient oncology appointments. Results were compared in identifying potential infectious agents. **Results**: BCs identified pathogens in only two patients, despite several cases where infection was suspected. In contrast, mcfDNA testing detected pathogens in 11 of the 14 patients, including bacteria, such as *Staphylococcus aureus*, and invasive fungi, such as *Candida* and *Aspergillus* species, and *Pneumocystis jirovecii*. **Conclusions**: In the outpatient setting, mcfDNA surveillance offers a more reliable method for detecting pathogens. This approach identified actionable microbiologic results in immunocompromised individuals who did not meet standard clinical criteria for suspicion of infection. Further research is required to confirm the potential of mcfDNA surveillance in an outpatient setting to guide more accurate treatment decisions, reduce extensive clinical investigations, and improve neutropenic patient outcomes.

## 1. Introduction

Acute myeloid leukemia (AML) is a hematologic malignancy that disrupts normal hematopoiesis and is linked to a high rate of mortality, often due to infectious complications. AML arises primarily from myeloid progenitor cells in the bone marrow [1] and progresses to bone marrow failure and organ infiltration [2]. The disease typically affects people over 60 years old, presents with symptoms that progress rapidly, and is fatal if not treated promptly and appropriately [3].

Neutropenia is a common and serious complication in patients with AML undergoing chemotherapy. Chemotherapy regimens for AML target rapidly dividing cells, including malignant cells and healthy hematopoietic progenitors in the bone marrow. As a result, the neutrophil count is suppressed, leading to severe neutropenia, traditionally defined by an absolute neutrophil count (ANC) of less than 0.5 K/μL (or 500 cells/μL) [4]. This state considerably increases the risk of life-threatening infections in these patients [5], making these individuals particularly vulnerable to infections, as their weakened immune system is not capable of efficiently fighting bacterial, viral, or fungal pathogens. Neutropenia accompanied by fever following induction chemotherapy occurs in more than 80% of cases [6] and often necessitates the use of antibiotics to mitigate infectious risk. Patients are susceptible to invasive infections, not infrequently from uncommon pathogens, and may manifest atypical signs and symptoms. Also, acute leukemia can mimic infectious symptoms, such as fever, fatigue and bone pain, making the diagnostic process lengthy and demanding for patients and clinicians [7].

For these reasons, various approaches have been employed to monitor the onset of infections in immunocompromised hosts. Standard culture-based diagnostic methods, such as blood cultures (BCs), widely used in hospital settings, show limited pathogen identification in these individuals due to low yields and often provide false-negative results [8]. Additionally, BCs have longer turnaround times and carry the risk of identifying contaminating microbes [9], particularly in emergency department settings [10]. This feature of BC could lead to additional laboratory testing, unnecessary treatments and prolonged hospital stay. Another diagnostic option is matrix-assisted laser desorption–ionization time-of-flight (MALDI-TOF) mass spectrometry, which is used in clinical laboratories and offers reduced processing times for identifying positive cultures. However, MALDI-TOF mass spectrometry still relies on BC for initial pathogen identification and has low specificity [11]. Diagnostic tests that rely on antibody responses are often unreliable in immunocompromised individuals who cannot produce adequate antibodies [12]. Moreover, biomarker-based tests may be influenced by sample type, host characteristics, antimicrobial exposure, or cross-reactivity [13]. Nucleic acid amplification-based technologies, including polymerase chain reaction (PCR), facilitate pathogen detection and analysis; however, their effectiveness depends on the bacterial load in the sample and the presence of PCR inhibitors [14]. While PCR tests are widely used for pathogen diagnosis, there is a need for standardization of PCR assays [15] and optimization of their sensitivity, as well as technical methodologies, including specimen type, testing frequency, and reporting timelines [16].

Plasma microbial cell-free DNA (mcfDNA) metagenomic sequencing represents a significant advancement in diagnosing infections in the inpatient setting, where the pathogen burden is often higher, and has gathered much interest among healthcare providers [17,18,19,20,21,22,23,24,25,26]. This method offers accurate, non-invasive, non-culture-based testing and allows fast and broad pathogen identification in hospitalized individuals who often present with atypical and non-specific symptoms or have multiple infections [12]. Various sequencing-based diagnostic approaches have been increasingly adopted in clinical settings to help prevent missed or incorrect diagnoses of infections; however, they present some limitations. These techniques often lack the sensitivity and breadth required to detect low pathogen levels or rare microbial species and may need standardization of bioinformatic protocols [27]. As a result, their reliability may be reduced, particularly in complex and challenging cases where diagnostic accuracy is critical.

This study aims to investigate whether plasma mcfDNA surveillance has the potential to provide similar advantages to an outpatient oncology population. To our knowledge, this application has not yet been explored in this specific context. We hypothesize that plasma mcfDNA metagenomic sequencing will benefit neutropenic AML patients in identifying potential pathogens with actionable management decisions.

## 2. Materials and Methods

This case series evaluates the clinical utility of plasma mcfDNA sequencing testing in neutropenic patients with newly diagnosed or relapsed/refractory AML undergoing outpatient chemotherapy. Through the analysis of 14 patient cases, this series demonstrates the practical application, sensitivity, and potential benefits of plasma mcfDNA surveillance in improving patient outcomes and reducing clinical investigations. The findings provide an insight into the potential of mcfDNA monitoring in an outpatient setting, laying the groundwork for broader studies to enhance the understanding and application of this technology across various clinical contexts.

The Karius Test (KT, Karius, Redwood City, CA, USA) is a non-invasive blood test that isolates mcfDNA while suppressing background human DNA from plasma. KT uses advanced bioinformatics to detect more than 1000 pathogens in circulating plasma. For each pathogen detected, a molecules-per-microliter (MPM) value is calculated, corresponding to the number of pathogen-specific mcfDNA molecules detected in one microliter of the patient’s plasma, thereby providing a quantitative estimate of that organism’s bloodstream burden. The KT is primarily designed to identify and diagnose infections caused by bacteria, DNA viruses, fungi, and parasites in patients [17,28], but it also has the ability to detect antimicrobial resistance genes, such as *SCCmec* and *mecA* for staphylococci and *vanA* for enterococci [29]. Previous studies have shown the clinical validity and utility in immunocompromised patients [18,19,20,21,22,23,24,25,26]. KT is performed in a Clinical Laboratory Improvement Amendments (CLIA)-certified/College of American Pathologists-accredited laboratory and can provide results in 18 h from sample receipt.

Recruitment of patients enrolled in this observational study was performed from December 2023 to November 2024 at the Massachusetts General Hospital (MGH) in Boston, MA, USA. A total of 15 patients diagnosed with AML who were undergoing outpatient chemotherapy at MGH consented to participate in the study, and 14 were ultimately enrolled. One patient (patient ID 3) was excluded because of fever prior to enrollment. Prior to study enrollment, all patients and/or their Legally Authorized Representatives provided written informed consent (IRB protocol #2023P002500). Blood samples were collected for mcfDNA metagenomic sequencing by KT up to two times per week at routine outpatient oncology follow-up appointments for approximately four months, stored frozen, and then batch analyzed retrospectively. After completion of all study visits, positive KT and pathogen identification results for each subject were compared to those from standard diagnostic methods to evaluate their effectiveness and potential impact on clinical approaches and patient outcomes. All patient information was de-identified and stored in a secure database for retrospective analysis, which was conducted after each patient’s completion of the study. These results were not disclosed to the physician care teams responsible for the patients, but an exception would have been made if a subject tested positive for a high-risk pathogen, defined by our protocol as one capable of resulting in devastating consequences for the individual and public health (e.g., *Mycobacterium tuberculosis*, *Plasmodium* spp., and *Varicella-zoster virus*).

Patients in this case series were selected based on criteria including a new or relapsed/refractory diagnosis of AML and the expectation to undergo outpatient chemotherapy resulting in neutropenia at MGH. Only those expected to continue treatment on-site and complete baseline KT within seven days of consent were considered. Exclusion criteria included individuals under 18 years of age, pregnant patients, and those with clinical, functional, psychosocial, or cognitive limitations that may impact follow-up compliance. Day zero marks the day of the subject’s consent; enrollment is defined as completion of baseline KT. The schedule of study visits was chosen to assess the clinical utility of outpatient mcfDNA surveillance when integrated into regular follow-up care. Study visits were defined as sample collection at 32 routine outpatient chemotherapy appointments, including no more than two appointments per week. Subjects completed their last study visit when one of the following criteria were met: completion of 32 study visits, two weeks after completion of chemotherapy, transfer of care outside of the enrolling site, or admission for suspicion of infection or febrile neutropenia. If the subject was admitted to the hospital for suspicion of infection, blood for the KT was collected on the day of admission and once more five days after admission, if applicable. End of study was defined as completion of a subject’s final study visit or procedure. Detailed information was extracted for each patient, including demographics, medical history, vital signs, and standard care data such as laboratory values, procedures, and microbiology tests. The study is exploratory, and hence analysis is mainly descriptive. Descriptive measures, including frequency and percent, were used to summarize the proportion of subjects with positive and negative test results. Confidence intervals for rates of infection were estimated using methods for exact binomial confidence intervals. McNemar’s test was used to compare the rate of infections detected between the BC and the KT. Time-to-event (infection) data were summarized using the Kaplan–Meier curves, with significance tested using the robust sandwich variance estimator of Wei, Lin, and Weissfeld [30]. All *p*-values are two-sided, and a *p*-value of less than 0.05 was considered significant. Statistical analyses were performed using the R package version 4.1.1 and SAS software version 9.4 (SAS Institute, Cary, NC, USA).

## 3. Results

**Figure 1:**
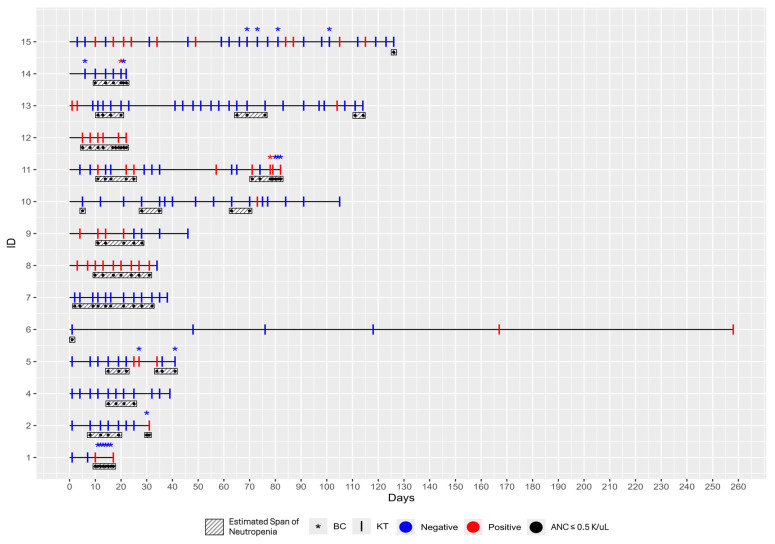
Duration of subject’s time in study (black line), time of KT (vertical lines), BC collection (asterisks), and their respective results (blue = negative; red = positive). Estimated span of severe neutropenia (black and white box) based on confirmed ANC ≤ 0.5 K/uL (black circle).

The 14 patients included in this case series were enrolled between 11 December 2023, and 1 November 2024. Their ages ranged from 24 to 83 years, with a median age of 61 (IQR = 44, 71). At the time of enrollment, thirteen patients had newly diagnosed AML, and one had relapsed AML. Treatments ranged from lower intensity chemotherapy, such as hypomethylating agents (HMA) + Venetoclax, to moderate and higher intensity treatments, including Vyxeos induction, 7 + 3 days of induction with cytarabine and an anthracycline, and high-dose cytarabine (HiDAC). Other treatments included quizartinib and dasatinib. Although not all patients were neutropenic at day zero, all developed neutropenia during the course of the study (Figure 1). Neutropenia was present for the majority of the study period (Table 1). None of the patients were receiving immunosuppressive therapy during the study period.

In this case series, the diagnostic performance of traditional BCs was compared to that of the KT in detecting infections among patients. BCs were required in 6 of the 14 patients during the study and 20 BCs were collected in total (Figure 1). Only two BCs, collected from two separate subjects, yielded significant findings and tested positive for pathogens, despite clinical suspicion of infection in all assessed patients. In particular, for Patient 11, four BCs were collected; the first turned positive within 15 h of collection, and *Streptococcus species* were identified at 40 h. For Patient 14, three BCs were collected; only the second one turned positive at 23 h of collection, with *Staphylococcus epidermidis* identified after 42 h. Urine cultures were also collected from these six patients, and only Patients 5 and 15 had a positive one. However, no pathogen was identified in Patient 5’s urine culture, whereas probable *Enterococcus* was reported in Patient 15’s sample. Patients 1 and 12 underwent a single galactomannan test, which yielded a negative result. Five individuals (Patients 1, 2, 11, 12, 14) were admitted to the hospital for febrile neutropenia or suspicion of infection, whereas two (Patients 5, 9) were admitted for non-infectious reasons.

In contrast, the KT demonstrated a significantly higher diagnostic yield. The KT identified mcfDNA from pathogenic organisms in 11 out of 14 patients (79%, 95% CI: 49–95%), while BCs were positive for only two out of the 14 patients (14%, 95% CI: 0–43% (Figure 1).

A broad spectrum of pathogens was detected by KTs during the study, with substantial variability in timing, microbial burden, and associated clinical findings (Table 2). Detected organisms included invasive fungi: *Aspergillus flavus*, *Aspergillus fumigatus*, *Aspergillus nidulans*, *Pneumocystis jirovecii*, *Candida parapsilosis*, *Candida albicans*, *Candida glabrata*, as well as bacterial pathogens such as *Staphylococcus aureus*, *Klebsiella pneumoniae*, *Pseudomonas aeruginosa*, *Bacteroides fragilis*, *Burkholderia cepacia complex*, *Enterocloster clostridioformis*, *Enterobacter cloacae*.

Patient 15 exhibited the highest pathogen diversity, with eight organisms detected, accompanied by respiratory symptoms and fatigue. High microbial loads were recorded in several patients, most notably Patient 11 with *P. aeruginosa* (MPM 1229) and Patient 13 with *Burkholderia cepacia complex* (MPM 1532), correlating with systemic symptoms such as fever, chills, cough and fatigue. Patient 8 also serves as an example of species-level identification clinical impact; four days before KT detected *P. jirovecii* (MPM 103 [21, 259]), the patient presented with worsening cough, dyspnea and lower extremity edema. At that time, chest CT imaging showed findings consistent with right lower lobe pneumonia. However, as there was no clinical suspicion for *P. jirovecii* infection, no additional diagnostic workup, including BAL, was performed. The patient was empirically treated with levofloxacin, amoxicillin/clavulanate (AMC) and fluconazole (FLC) which did not provide appropriate coverage for *P. jirovecii*. As a result, symptoms such as exertional dyspnea, nocturnal coughing, and vomiting persisted for nearly a month. Similarly, in Patient 15 and Patient 9, no specific diagnostic evaluation for aspergillosis was performed, as the clinical symptoms were nonspecific. Patient 15 developed congestion, cough, fatigue and dyspnea on exertion, while Patient 9 experienced nausea, vomiting, fatigue and a rash during follow-up. In both cases, no clinical suspicion for invasive aspergillosis was raised. Nevertheless, KT was able to detect *Aspergillus* spp. in both patients.

Across the cohort, detection times and the end of the study (EOS) prophylactic regimens varied, with agents such as acyclovir (ACV), fluoroquinolones (FQ) and antifungals like posaconazole (PCZ) frequently administered. This heterogeneity underlines the complexity of microbial exposures and clinical management in AML patients. Of note, the KT accurately detected low-abundance mcfDNA, providing targeted results.

**Figure 2:**
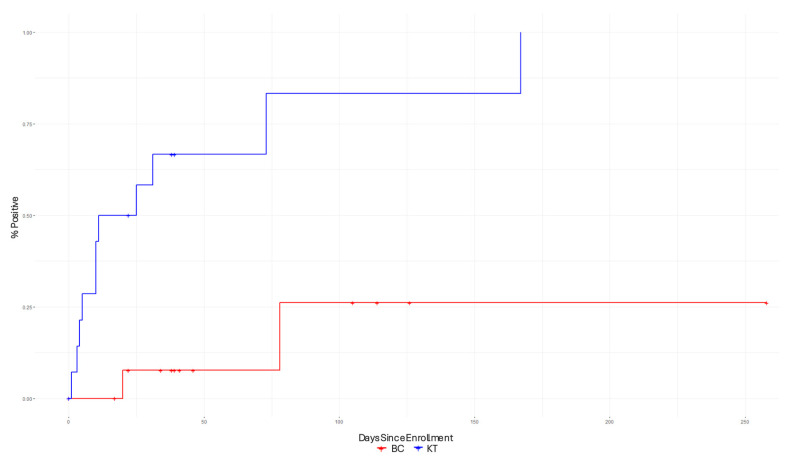
Kaplan–Meier curves demonstrate the improved diagnostic performance of the KT compared to conventional culture-based tests (*p*-value < 0.001).

Figure 2 presents the Kaplan–Meier curves, which were used to compare the time-to-pathogen detection between the BCs and KTs. The Kaplan–Meier curves show that the KT had shorter time-to-pathogen detection compared to the BC.

After the end of the study and their final KT, all 14 patients were monitored for four months in the electronic medical record system. At the end of this period, thirteen patients were still attending their scheduled outpatient clinic follow-up visits, whereas one patient was deceased a month after their last KT.

## 4. Discussion

This case series highlights the utility of the KT for noninvasive mcfDNA-based pathogen detection in neutropenic AML patients undergoing outpatient chemotherapy. KT identified critical pathogens in 11 of the 14 patients, outperforming traditional BCs, which only provided positive results for two patients. Various pathogens were detected, including bacteria and fungi, such as *Pseudomonas aeruginosa*, *Aspergillus flavus*, *Aspergillus fumigatus* and *Pneumocystis jirovecii*. These fungal species are known to cause serious, potentially life-threatening infections, particularly in individuals with weakened immune systems [31,32]. The findings demonstrate the clinical significance and high diagnostic range of the KT, highlighting its potential to improve diagnostic accuracy across a wide range of clinical applications.

Despite their widespread use, traditional BCs often present limitations, such as delayed turnaround times and low diagnostic yields, particularly for pathogens present at low levels in the bloodstream. They also do not provide immediate information about the pathogen’s resistance to antibiotics, as susceptibility testing is typically performed after culture growth. Remarkably, KT delivers actionable results in approximately 18 h, while traditional BCs typically require 24–72 h for most bacterial and yeast pathogens and may take longer for slower-growing organisms [33,34,35]. This turnaround time is critical since the extended lengths of hospital stays have been linked to delays in processing and appropriately treating bloodstream infections [36].

The KT offers clear advantages over conventional methods, especially in neutropenic patients, where early and accurate infection detection is crucial for optimal outcomes. However, it also presents some limitations, particularly its heterogeneity in performance across different pathogen groups. While its performance is greater for detecting bacterial and invasive non-*Aspergillus* mold infections, its sensitivity to *Aspergillus* infections remains less robust [25]. In our study, multiple *Aspergillus* species were detected in subject nine, which may suggest improved sensitivity (Table 2).

In summary, while KT offers clear advantages in terms of rapid and broad pathogen detection, both KT and BCs present limitations. Nevertheless, BCs still remain valuable for guiding treatment, given their specificity and predictive value for bloodstream infections in neutropenic patients [37].

By merging KT and clinical data, physicians have the potential to make more targeted treatment decisions in the outpatient setting. This combination could help automate antimicrobial recommendations, improving efficiency and consistency in infection management. The KT also has the potential to optimize resource utilization by reducing unnecessary tests, preventing hospitalizations, and minimizing healthcare costs. As a result, this would support a more efficient healthcare system, and patients would benefit from more effective care and faster recovery times.

To date, the performance and value of the KT have focused on the inpatient setting, where pathogen levels are typically higher and patients are at greater risk of acquiring infections [38,39]. Hospitalized patients are exposed to significant nosocomial flora and require the use of medical devices, such as venous or urinary catheters, endotracheal tubes, and surgical drains. Through these exposures, inpatients often experience more serious infections with higher microbial burdens [40]. A larger challenge has been the identification of potential pathogens in patient cohorts at risk, but currently in a subclinical state without current symptoms.

In this study—the first to our knowledge to apply plasma mcfDNA sequencing testing prospectively in an ambulatory oncology cohort—we show that mcfDNA detection methods, such as the KT, may serve as a potential tool to identify clinically relevant pathogens in asymptomatic, clinically stable outpatients. Until now, it was unknown whether subclinical pathogen burdens could be captured outside of the inpatient setting, or whether such signals would correlate with subsequent hospital admission. We demonstrate that appreciable microbial loads can be present even in patients who are well enough to forgo invasive diagnostic procedures, typically avoided to minimize the risk of pathogen transmission. However, those microbial burdens may still precipitate serious, potentially life-threatening complications if left unidentified. Future studies with larger cohorts should incorporate confirmatory diagnostics, such as MALDI-TOF and other ancillary microbiological tests, to more accurately assess the true and false positivity rates of mcfDNA results and strengthen clinical interpretation. Nevertheless, these data highlight the need for a prospective, placebo-controlled interventional trial to establish how far in advance of a potential clinical deterioration these pathogens can be detected by the KT and whether species-directed therapy improves outcomes compared with current standard care.

This is a single-center case series study, so several limitations should be considered, including a small sample size of 14 subjects, a retrospective design, a lack of a gold standard for the KT, and batch analysis of samples. Additionally, selection bias may have influenced the results, as the participants may not represent the broader population of patients with AML. Differences in age, disease stage, and chemotherapy regimens may also influence findings. Participants were limited to a maximum of two tests per week, but due to clinical challenges, some patients had longer intervals between KTs. Despite these limitations, we provide valuable initial findings that should support further investigation into the use of plasma mcfDNA metagenomic sequencing for pathogen detection in neutropenic patients in the outpatient setting.

## 5. Conclusions

In summary, our prospective case series is the first to introduce the KT to the outpatient oncology setting and evaluate its utility for detecting potential infectious pathogens in chemotherapy-treated neutropenic patients with AML, who show no critical clinical signs of infection (e.g., no major systemic symptoms). The KT outperformed traditional BCs, demonstrating higher sensitivity and its potential as a more precise, species-directed therapy. Our data show that plasma mcfDNA surveillance could have a role in ambulatory care to detect clinically significant and potentially harmful pathogens that would otherwise remain undetected. These data suggest that a randomized, KT-guided interventional clinical trial is warranted to confirm the potential for optimizing resource utilization in the outpatient setting, guiding treatment decisions, and enhancing outcomes in neutropenic outpatients.

## Figures and Tables

**Table 1:** Demographics and medical history.

Patient ID	Age (y)	Sex	Race	Hematologic Malignancy	Treatment	ANC Day 0	Percent of Study Days with Neutropenia (%)
1	60	M	White	AML (ND)	7 + 3 induction, Quizartinib, HiDAC	6.02	50%
2	69	M	White	AML (ND)	HMA + Venetoclax	1.56	61%
4	51	F	Asian	AML (ND)	7 + 3 induction, Quizartinib, HiDAC	2.92	50%
5	76	M	White	AML (ND)	HMA + Venetoclax	1.55	60%
6	63	M	N/A	AML (ND)	HMA + Venetoclax	0.21	10%
7	24	M	White	AML (ND)	7 + 3 induction, Quizartinib, HiDAC	0.09	92%
8	71	M	White	AML (ND)	7 + 3 induction, Quizartinib, HiDAC	2.04	71%
9	61	F	White	AML (ND)	7 + 3 induction, Quizartinib, HiDAC	2.57	50%
10	72	M	White	AML (ND)	HMA + Venetoclax	0.36	20%
11	44	M	White	AML (Relapsed)	HiDAC + Dasatinib	2.71	41%
12	32	M	White	AML (ND)	HMA + Venetoclax, Vyxeos Induction	0	100%
13	27	F	N/A	AML (ND)	HiDAC	4.9	24%
14	83	M	White	AML (ND)	HMA + Venetoclax	0.71	88%
15	61	F	White	AML (ND)	HMA + Venetoclax	2.7	2%

ND = new diagnosis; ANC = absolute neutrophil count (neutropenia ANC < 0.5 K/uL); Day 0 = day of baseline KT; 7 + 3 induction = 7 + 3 days of induction with cytarabine and an anthracycline; HMA = hypomethylating agent; HiDAC = high-dose cytarabine.

**Table 2:** Pathogens detected by the KT, timing of detection, microbial load, clinical findings and prophylaxis at the end of study.

Patient ID	Pathogen Detected by KT	Time of Detection by KT (Day of Study)	MPM, Mean [Range]	Objective Findings at EOS	Prophylaxis at EOS
15	*Stenotrophomonas maltophilia*	Day 11	214	Cough, Dyspnea on exertion, Fatigue	ACV
	*Corynebacterium xerosis*	Days 11–21	130 [104, 153]		
	*Rhodococcus fascians*	Days 14–21	65.7 [59, 72]		
	*Brucella anthropi*	Day 14	19		
	*Human herpesvirus 7*	Days 31, 81–84, 102	10.7 [10, 12]		
	*Aureobasidium pullulans*	Day 46	22		
	*Staphylococcus pettenkoferi*	Day 46	20		
	*Aspergillus nidulans*	Day 112	<10		
14	None detected	N/A	N/A	Diarrhea, Lightheadedness	ACV
13	*Human adenovirus D*	Days 0–2	105	Cough, Congestion, Fatigue	AUG, FQ, ACV
	*Burkholderia cepacia complex*	Day 103	1532		
	*Klebsiella pneumoniae*	Day 103	982		
12	*Candida albicans*	Days 0–17	70.5 [20, 234]	Cough, Congestion, Fatigue, Headaches, Sinus pain	AUG, FQ, ISA, RDV, ACV
	*Enterobacter cloacae complex*	Day 0	172		
	*Malassezia globosa*	Day 14	32		
	*Staphylococcus haemolyticus*	Day 14	12		
	*Cupriavidus metallidurans*	Day 17	113		
11	*Pseudomonas aeruginosa*	Day 7	1229	Fever, Chills, Diarrhea, Fatigue, Wheezing, Dizziness	DOX, VAN, PTZ, FCV, RDV, PCZ
	*Stenotrophomonas maltophilia*	Days 7, 67	485 817		
	*Candida glabrata*	Days 18–21	18.5 [18, 19]		
	*Bacteroides fragilis*	Day 53	74		
	*Streptococcus infantis*	Days 74–78	656 [33, 1691]		
	*Streptococcus peroris*	Days 74–78	250 [17, 593]		
10	*Corynebacterium jeikeium*	Day 68	64	Fatigue	None
9	*Aspergillus flavus*	Days 0–10	17.7 [10, 29]	Tooth pain	CLI, MCF
	*Aspergillus fumigatus*	Days 7–10	11 [10, 12]		
	*Streptococcus mitis*	Day 17	3023		
8	*Pneumocystis jirovecii*	Days 0–28	103 [21, 259]	Cough, Dyspnea, Lower extremity edema	AUG, FQ, FCZ, ACV
7	None detected	N/A	N/A	None observed	ACV
6	*Aureobasidium pullulans*	Day 166	148	Decreased appetite	ACV
	*Auerobasidium melanogenum*	Day 166	214		
	*Clostridium perfrigens*	Day 257	18		
	*Enterocytozoon bieneusi*	Day 257	368		
5	*Cutibacterium namnetense*	Day 24	710	Fever, Chest pain	FEP, ACV
	*Porphyromonas gingivalis*	Day 24	93		
	*Staphylococcus aureus*	Days 26–33	75.5 [50, 101]		
4	None detected	N/A	N/A	None observed	ACV
2	*Enterocloster clostiridioformis*	Day 30	74	Fever, Chills	FEP, MTZ, ACV
	*Enterobacter cloacae complex*	Day 30	103		
1	*Candida parapsilosis*	Days 9, 16	38 [11, 65]	Cough, Congestion, Fatigue, Headaches, Sinus pain	FEP, ACV, MCF

MPM = molecules per Microliter; EOS = end of study; N/A = not applicable; FEP = cefepime; ACV = acyclovir; FLC = fluconazole; MTZ = metronidazole; MCF = micafungin; CLI = clindamycin; RDV = remdesivir; PTZ = piperacillin-tazobactam, Zosyn; DOX = doxycycline; ISA = isavuconazole; FCV = famciclovir; VAN = vancomycin; PCZ = posaconazole; AMC = amoxicillin-clavulanate, augmentin.

## Data Availability

Data are available from the corresponding author.
